# Smoking Prevalence and Secondhand Smoke Exposure during Pregnancy and Postpartum—Establishing Risks to Health and Human Rights before Developing a Tailored Programme for Smoking Cessation

**DOI:** 10.3390/ijerph17061838

**Published:** 2020-03-12

**Authors:** Kate Frazer, Patricia Fitzpatrick, Mary Brosnan, Anne Marie Dromey, Sarah Kelly, Michael Murphy, Denise O’Brien, Cecily C. Kelleher, Fionnuala M. McAuliffe

**Affiliations:** 1 School of Nursing, Midwifery and Health Systems, Belfield, Dublin 4, Ireland; 2 School of Public Health, Physiotherapy and Sports Science, University College Dublin, Woodview House, Belfield, Dublin 4, Ireland; 3 Department of Preventive Medicine & Health Promotion, St Vincent’s University Hospital, Elm Park, Dublin 4, Ireland; 4 National Maternity Hospital, Holles Street, Dublin 2, Ireland; 5 UCD Perinatal Research Centre, School of Medicine, University College Dublin, National Maternity Hospital, Dublin 2, Ireland; 6 University College Dublin, College of Health and Agricultural Sciences, Woodview House, Belfield, Dublin 4, Ireland

**Keywords:** secondhand smoke, smoking, pregnancy, public health, tobacco control, human rights

## Abstract

Both smoking during pregnancy and secondhand smoke exposure are associated with reduced health outcomes. However, limited consistent evidence exists of risks of secondhand smoke exposure in pregnancy. Currently, inadequate smoking cessation services exist in Irish maternity hospitals. To identify the number of pregnant women smoking during pregnancy and to identify their exposure to secondhand smoke, we conducted a cross-sectional observational pilot study in one regional maternity hospital in Ireland in July/August 2018. Respondents were (1) women attending antenatal clinics and (2) postpartum women before discharge. Variables measured included smoking status of pregnant women and partner status, demographic variables, secondhand smoke exposure, and support for hospital smoke-free policy and development of smoking cessation services. The overall response rate was 42.2% in this study. The response rate was 56.5% (111/196) from postnatal wards and 37.3% (215/577) from antenatal clinics. Over 40% of respondents reported they had smoked during their lifetime. The majority of women (70%) reported quitting smoking before their pregnancy. Few women were active smokers. Almost 40% reported exposure to tobacco smoke in the previous week (38.5%); 16.9% reported living with a smoker, a critical factor in increased risk (Odds Ratio (OR) 3.89, 95% CI = 1.86–8.15, *p* < 0.001). Approximately 10% of postnatal mothers reported that their newborn would travel home with a smoker. Support for a no-smoking hospital policy was very high as was support for the development of cessation services. No documentation of secondhand smoke exposure for pregnant women or newborns is sought or recorded routinely in the hospital. A systems approach to develop smoking cessation programmes in maternity care should include screening and documenting of secondhand smoke exposure risks for women during pregnancy, and for their newborns at discharge, to improve health outcomes and protect human rights.

## 1. Introduction

Over 8 million deaths worldwide are associated with smoking annually; seven million associated with active smoking and 1.2 million deaths due to exposure to secondhand smoke (SHS) [1]. There is no safe level of SHS exposure, and Ireland has led internationally as the first country to introduce smoke-free legislation banning smoking indoors in public places in 2004 [2]. A 2014 report of the US Surgeon General identified 400,000 live-born infants exposed in utero to tobacco from maternal smoking annually [3,4,5] and found that women who are more likely to smoke are those already disadvantaged by low education and low income. Overall these women are less likely to quit smoking when pregnant and are more likely to relapse after delivery [3,6]. Ultimately reducing the prevalence of smoking among pregnant women is an international public health goal. Evidence-based population measures to reduce exposure to tobacco smoke include the Framework Convention on Tobacco Control (FCTC) and MPOWER initiatives [7,8,9]. Key population groups benefiting from the enactment of legislative smoking bans are pregnant women, children, and non-smokers [2,9].

Smoking is a modifiable risk factor during pregnancy and reported risks are ectopic pregnancies, placenta praevia, pre-eclampsia, and reduced foetal outcomes including mortality, low birth weight, and sudden infant death syndrome [10]. Banderali’s review reported evidence for the harmful effects of foetal and postnatal exposure to paternal smoking, including preterm birth, low birth weight, evidence of risk of obesity, and risk of hypertension in later life [11]. Additional adverse effects of SHS exposure during pregnancy in non-smoking women includes the risk of stillbirth and congenital malformations [12]. This evidence supports the Barker hypothesis and life course epidemiological approach emphasizing the importance of the first 1000 days after conception. Maternal smoking during pregnancy plays a significant role in adverse postnatal outcomes, and it may cumulate negatively with SHS exposure [13,14].

Similarly, Kabir reported reduced maternal smoking and reductions in preterm births following the introduction of legislative smoking bans in Ireland, and evidence that nicotine adversely affects maternal and foetal health during pregnancy, leading to reduced health outcomes [15]. Evidence of SHS exposure in Ireland in pregnant non-smokers is limited, and no published Irish data on SHS exposure rates during pregnancy existed when the current study commenced. Subsequently, Reynolds reported a passive smoke exposure level of 28.0% for pregnant smokers and 15.9% for pregnant non-smokers attending a maternity hospital [16]. Do [17] acknowledges limited evidence of SHS for these women despite the public health consequences. While Ngo reports SHS exposure rates in Vietnam of over 90% among pregnant women, other international comparisons range from 20% to 79%, but the included studies were carried out before 2010 [18]. Bloch reports SHS exposures internationally from 6.1% to almost 80% [19]; however, Siddiqi acknowledges a lack of evidence and the need for more studies [20].

Smoking cessation practices are fundamental for all health care professionals’ interactions, as unequivocal evidence acknowledges that smoking cessation improves life expectancy and reduces the risk of chronic illness [1,21,22,23]. The year 2015 overlapped with a 10-year review of the FCTC and an introduction of UN Sustainable Development Goals (SDGs) targets for 2030 [24]. While tobacco control is a critical element to achieve worldwide success for SDGs, the World Health Organization (WHO) concede that there is slow progress in many countries and a need for improved sustainable infrastructure and widening of access to cessation supports [1,24].

Nationally, Tobacco Free Ireland aims to reduce smoking prevalence to <5% by 2025, pursuing key initiatives and recommendations, including widening legislation in support of international evidence [1,25]. Current reports identify a reduction in national smoking rates to 17% overall, but higher for parents (19%) and in those living in deprived areas (40%) [26]. A national maternity strategy highlights the absence of onsite smoking cessation services and training for midwives and health care professionals; therefore, opportunities exist for developing services and reducing risks from smoking [27]. Published smoking prevalence rates during pregnancy were found to be 18% in a national cohort study [28]. Variations in smoking rates range from over 40% in women identified from lower socio-economic groups to less than 10% smoking rates in women recorded as belonging to higher socio-economic groups [28]. More recently, Reynolds reported 11% smoking prevalence in an audit of all 19 Irish maternity units; and subsequently, in a study including the measurement of carbon monoxide levels, identified an underreporting of active smoking by 40% [16,29]. These studies confirm the extent of the Irish problem and variation in the smoking cessation services offered to pregnant women. A single maternity unit screens and measures carbon monoxide levels; whereas this practice is routine in Scotland where smoking rates in pregnancy are 29.3% for those confirmed as living in deprivation [30].

The Irish Tobacco Free Implementation plan incorporates maternity care services including the introduction of carbon monoxide monitoring, Make Every Contact Count [MECC] training for all employees, and introducing a targeted communication campaign for promoting smoke-free cars (p. 18) [31]. Reynolds demonstrates gaps in service provision in Ireland for pregnant women despite national and international strategic policies advocating for smoking cessation services during pregnancy [29].

The impact of SHS exposure during pregnancy is unknown; partner smoking habits are undocumented despite clean air accepted as a fundamental human right [7]. Smoking habits recorded at first antenatal visits in Ireland identifies the smoking status of pregnant women, but do not report risks of SHS exposure before or during pregnancy; these are components of international NICE guidelines [32].

The WHO guidance on tobacco use and SHS exposure during pregnancy provides direction for developments to support pregnant women internationally and nationally [33]. Irish advancements in smoking cessation services in pregnancy are long-awaited; Lange reported estimated global prevalence of smoking during pregnancy as 1.7% (95% CI 0.0%–4.5%) globally, 8.1% in European region (95% CI 4.0%–12.2%) and Ireland specifically at 38.4% (95% CI 25.4%–52.4%) [10]. Frazer reported limited consistent evidence identifying the impact of national smoking bans on perinatal health outcomes internationally [2]. National prevalence data for SHS exposure during pregnancy in Ireland are unknown and limited details explaining the risks exist.

This study aimed to identify smoking prevalence rates for pregnant and postpartum women and detail their exposure to SHS in one tertiary level maternity hospital with circa > 8000 deliveries annually before developing and instituting a smoking cessation programme. The study objectives were:

1. To identify smoking prevalence rates in pregnant women attending antenatal clinics and in the postpartum period before discharge.
2. To identify the secondhand/passive smoke exposure of pregnant women attending antenatal clinics and in the postpartum period before discharge.
3. To measure the quit attempts identified by women during pregnancy.
4. To identify smoking cessation services offered to women during pregnancy.

## 2. Materials and Methods

### 2.1. Study Design

This was a pilot study using a prospective cross-sectional survey design with institutional ethical approval and consent from women. Data were collected using a modified survey tool, and details reported following the STROBE criteria [34].

### 2.2. Ethical Approval

Ethical approval was obtained from the hospital’s Human Ethics Committee and included a review of all documentation, including a patient consent form, patient information leaflets, and study-specific instrument (EC12.2018).

### 2.3. Data Collection

Pregnant women attending antenatal clinics in one Dublin maternity hospital were recruited during a one-week data collection period (July 2018) and invited to participate. A study pack comprising an information leaflet, consent form, and questionnaire was distributed to all pregnant women (who met the inclusion criteria of aged ≥18 years, understand English, written consent).

Similar data collection procedures were employed in all three postnatal wards in the maternity hospital. Postpartum women who met the inclusion criteria (written consent and following uncomplicated birth) were invited to participate and provided with a survey pack. Data were collected over two weeks (July/August 2018).

Research assistants were present to answer queries and assist with data collection. Completed questionnaires and signed consent forms were returned to collection boxes in antenatal clinics and ward locations. All questionnaires were anonymous and did not ask for personally identifying data.

#### Sample and Sample Size

There are 19 maternity hospitals in Ireland; the national birth rate in 2018 was 61,000 [35]. Our sample consisted of women aged 18 years and older attending one tertiary ^1^ level maternity hospital with >8000 deliveries annually. Non-randomized sampling procedures were adopted in line with ethical approval. Hospital data estimates identified that approximately 100 women attend antenatal clinics daily, and that approximately 20 women are discharged daily from postnatal wards. This was a pilot study, in advance of developing a smoking cessation service, and all women attending antenatal clinics and all women registered in three postnatal wards (who met the inclusion criteria) were invited to maximize response rates. The period for data collection was limited due to the availability of personnel.

### 2.4. Survey Instrument

A self-report study-specific tool was used for data collection. A survey instrument used previously in one general tertiary hospital over several years was accessed and modified for a maternity hospital setting [36,37]. Questions sought data on demographics, smoking habits during pregnancy (if the subjects ever smoked during their life, stopped before or during pregnancy, reduced smoking or no change in smoking status during pregnancy), quit attempts, SHS exposure, and use of smoking cessation services. Additional questions for postpartum women included identification of SHS exposure at discharge. Other items included in the study instrument sought information on support for the smoke-free hospital policy and the development of smoking cessation services.

### 2.5. Statistical Analysis

Bivariate analysis using Pearson’s Chi-squared and Fisher’s Exact Tests compared proportions, and a two-sample t-test was used to compare means in independent groups. Multivariate analyses, including logistic regression modelling, were carried out using discretionary forward elimination for exposure to SHS (Y/N). Variables selected for inclusion in the model were demographics. Age (continuous variable) was included, as previous studies suggest younger-aged women are at risk. Education status (two groups primary/post-primary V third level) and registration status (two groups public V private/semi-private) were included as proxy variables for a socio-economic measure. The risk of SHS exposure is higher for those living in deprivation. Finally, the variable “living with a smoker” (two group Y/N) is a proxy measure for SHS exposure in the home. Analysis using smoking status is omitted as few smokers participated in the study. Statistical significance was at the 0.05 level. Exact 95% confidence intervals were calculated for regression adjusted odds ratios. Data analyses were undertaken using SPSS V.24 (IBM Corp., Armonk, NY).

## 3. Results

### 3.1. Response Rate

The overall response rate was 42.2% in this study. The response rate was 56.5% (111/196) from the postnatal wards, and the response rate was 37.3% (215/577) from the antenatal clinics. Four questionnaires were excluded from the overall analysis due to extensive missing data; subsequent data analyses report 322 questionnaires.

### 3.2. Demographic Profile

The majority of respondents were registered as public patients and the mean duration of pregnancy at the time of data collection was 30.3 weeks (SD 8.8); with a range of 6 to 42 weeks (Table 1). The mean age of respondents was 33.4 years (SD 4.6), a sizable majority were multigravidae, a majority were employed, and over 85% of respondents had attained third level education (Table 1). In total, 188 women stated that they had other children living at home ranging from 1 to 5 (AN Mean 1.4 SD 8.8; PN Mean 1.6 SD 3.7).

### 3.3. Smoking Prevalence and Quit Attempts

Over 40% of respondents identified they had ever smoked during their lifetime. In the antenatal clinics, this accounted for 43.3% of respondents and over one-third of respondents in postnatal wards (34.3%) (Table 2). A majority of respondents reported they had stopped smoking before pregnancy and had not smoked since. The analysis of this subgroup identified almost 70% quit smoking before pregnancy, and a further 22% stopped smoking when they were aware of pregnancy and less than 10% of respondents self-reported active smoking (Table 2). In addition, 8 of the 11 smokers reported that they would like to quit smoking and would like assistance quitting. Due to the small sample size, there was no further analysis completed. Finally, less than 10% of respondents had tried vaping (electronic cigarettes) (Table 2).

### 3.4. Secondhand Smoke Exposures

A sizable minority (38.5%) reported exposure to SHS in the previous week, and almost 17% identified living with a smoker. Analysis identified partners/spouses as the primary smoker in the home (88.2%) (Table 3). A majority of all respondents reported having a smoke free area in their homes; less than 10% of postnatal mothers identified that their newborn would be transported home with a smoker. Almost 96% of respondents were aware of the harm of passive smoke exposure (95.7%) (Table 3).

Data from one open question enabled respondents to provide more detail and identify recent locations and situations of when they were exposed to SHS. Responses were grouped, and three areas of SHS exposure were identified. Firstly, personal interactions, including within their own home, with neighbours, visiting family and friends, or when attending family social events (weddings or funerals). Secondly, transport and locations when waiting at bus stops, on train platforms (Luas/DART stations), walking in the streets, when entering shops, and waiting at traffic lights. Finally, respondents identified social engagements, including attending outdoor concerts, visiting parks, and accessing children’s outdoor playgrounds. A source of SHS exposure identified by women was at the entrance to the hospital, particularly when attending antenatal appointments. The comments acknowledged the presence of smokers at entrance points.

### 3.5. Access to Smoking Cessation Service and Knowledge of Hospital Policies

Over 75% of women indicated that the hospital should provide smoking cessation supports (Table 4). A majority of respondents reported their awareness and support for the hospital’s smoke-free policy.

### 3.6. Regression Analysis

Multivariate logistic regression analysis was performed to identify independent demographic factors identified from literature that could explain an increased risk of SHS exposure (Table 5). Controlling for variables (age, public or private registration status, education level attained, and living with a smoker) pregnant women were over almost four times more likely to be exposed to SHS when living with a smoker (Odds Ratio (OR) 3.89, 95% CI = 1.86–8.15, *p* < 0.001.

## 4. Discussion

Development of smoking cessation services in maternity care are at a planning stage nationally, and the current study provides a focus on broader social norms to include women and partners/others at home who smoke. This study identified that approximately 10% of women were active smokers; a majority report quitting smoking before or when they are aware of their pregnancy. Smoking cessation services need to develop beyond a sole focus on pregnant women and consider and manage the impact of SHS exposures on foetal growth and development. Others control the environment of a child, and the human rights of children to health and wellbeing must be considered in the planning and rolling out of planned smoking cessation services, including in maternity care [38,39].

The results from this prospective observational study completed in advance of the development of smoking cessation services identify SHS exposure for 38.5% of respondents; living with a smoker is a critical risk factor. This is an undocumented risk. Assessment of smoking status and SHS exposure in maternity care services should include biochemical monitoring at appointments; carbon monoxide testing should be usual practice and is part of a future national implementation plan in Ireland [31]. The NICE guidelines [32] seek carbon monoxide testing in maternity care services and the latest developments in practice include an opt-out referral to smoking cessation services. At the time of this study, no screening programme was in place, and the NICE guidelines were published later, in 2019. Comprehensive approaches and initiatives are providing evidence of improved results [40,41] and the importance of adopting initiatives to target those most at risk.

The risks to adults and children from smoking are acknowledged [1,2]. Results from the current study provide further evidence for developing a systems approach to smoking cessation services in maternity care; this approach starts peri-conceptually and antenatally [13,42]. In the current study, almost 10% of mothers reported their children would travel home with a smoker, again highlighting a risk factor for SHS exposure that is undocumented or discussed. A majority of respondents were multigravidae (up to five children); therefore, increasing SHS exposure beyond a pregnant woman and suggesting opportunities for adopting teachable moments [33,43]. Faber (2019) reported risks for children exposed to SHS when in cars [38]. While Irish smoke-free legislation limits smoking in cars when children are passengers, there is sparse data identifying the enforcement of this legislation.

National data suggest 40% of the Irish population have tried to quit smoking in the past year, and a further 28% are trying or considering quitting. However, only 7% of successful quit attempts involve a health professional, and 52% of successful quit attempts did not use interventions or replacement therapies [26]. Quit rates in Ireland are less than those reported internationally [30,44]. What is evident is that 25% of quit attempts are successful, and there are opportunities to support specific population groups to quit as 33% of parents report trying to or planning to quit smoking [26].

Staff education is an essential component of smoking cessation programmes and results in active engagement with smokers attending health care services [21,23], and should be fundamental in maternity care [27,31]. Campbell’s study reiterates the importance of staff training in managing queries and engaging with pregnant women [40]. Their results suggest it leads to reductions in concerns and disengagement among staff due to their misplaced concerns about offending pregnant women. In their subsequent review of behavioural change techniques, Campbell acknowledges a woman’s desire to protect their child is an essential facilitator in supporting quitting during pregnancy. They suggest using potentially effective behaviour change techniques, including the value of credible experts to deliver the interventions and tailoring information to a woman’s circumstances [41].

Women in the current study supported the hospital’s smoke-free policy; a sizeable majority was in favour of developing smoking cessation services. Respondents did report exposure to SHS at the entrance to the hospital despite national policy implementing smoke-free campuses in hospitals (including no smoking at entrances and car parks) since 2013 [25]. Pregnant women identified smokers present at the entrance points to clinics in contrast with the hospital policy. Following the completion of this study, the Director of Midwifery was instrumental in seeking engagement with a national funding body and, in line with national policy, a smoking cessation service is being established in the hospital in 2020 [45].

Several limitations are acknowledged and are worthy of consideration. This was a cross-sectional pilot study and the time frame for data collection was brief due to the availability of personnel to assist with data collecting; this may have impacted the final response rate. Participation in the study was not mandatory; those who participated were self-selecting which resulted in a sample comprising women who were employed, highly educated, and not currently smoking. Other studies completed in this hospital have reported similar study demographics [46,47]. The number of current smokers in the study was less than 10%; smoking status was not verified and may be underreported. Despite efforts to recruit women to participate in the study, data are limited from current smokers and those not educated to the third level or who may be unemployed. Engagement with smoking cessation services is lower among those who experience higher levels of deprivation [30], and alternative research methods are required to engage actively with these population groups attending maternity services.

However, the results identify women self-reporting smoking during pregnancy and a continued risk of SHS exposure for those attending maternity care. There is an urgent need for the addition of smoking cessation services. Results confirm no documentation of partners’ smoking status despite the risk for pregnant women. In this study, 10% of babies travel home with a smoker—this risk is not identified or documented in maternity hospital records. This study adds to a growing body of evidence that identifies active smoking and SHS exposure across the course of life.

## 5. Conclusions

Despite Ireland’s role internationally in implementing a legislative smoking ban in 2004, restricting smoking indoors in public places and supporting the human right to clean air, there remain populations at risk during pregnancy. Enforcing smoking bans and policies and supporting smokers and their partners during pregnancy are elements of a systems approach to tobacco-free initiatives. Pregnant women in Ireland are at risk of SHS exposure. There is a critical need to develop responsive smoking cessation services for pregnant women and their partners; the evidence is unambiguous.

## Figures and Tables

**Table 1:** Demographic profile of respondents.

	Total		Antenatal (AN)		Postnatal (PN)		Significance (*p* < 0.05)
	Frequency	%	Frequency	%	Frequency	%	
Number weeks pregnant			Mean (SD) 30.3 (8.8)		N/A		
			Range 6 weeks to 42 weeks				
Hospital registration	*N* = 320		*N* = 210		*N* = 110		
Public	251	78.4	202	96.2	49	44.5	*p* = 0.001
Semi-private	45	14.1	7	3.3	38	34.5	
Private	24	7.5	1	0.5	23	20.9	
First pregnancy	*N* = 320		*N* = 211		*N* = 109		
Y	129	40.3	96	45.5	33	30.3	*p*= 0.009
N	191	59.7	115	54.5	76	69.7	
Age (years)	(*n* = 296) Mean (SD)		(*n* = 194) Mean (SD)		(*n* = 102) Mean (SD)		*p* = 0.03
	33.4 (4.6)		32.8 (4.9)		34.4 (3.7)		
	Range 19 to 47		Range 19 to 47		Range 23 to 45		
Employment	*N* = 320		*N* = 211		*N* = 109		
Paid	245	76.6	159	75.4	86	78.9	*p* = 0.374
Unpaid	4	1.3	3	1.4	1	0.9	
Self-employed	18	5.6	10	4.7	8	7.3	
At home	29	9.1	19	9.0	10	9.3	
Not working	18	5.6	16	7.6	2	1.8	
Other (including student)	6	1.8	4	1.9	2	1.8	
Highest Education	*N* = 316		*N* = 208		*N* = 108		
Primary	3	0.9	3	1.4	0	0	*p* = 0.028
Post-primary	40	12.7	29	14.0	11	10.2	
Third level	273	86.4	176	84.6	97	89.8	

**Table 2:** Current smoking status of pregnant women and mothers.

	Total		Antenatal (AN)		Postnatal (PN)		Significance (*p* < 0.05)
	Frequency	%	Frequency	%	Frequency	%	
Smoking status	*N* = 318		*N* = 210		*N* = 110		
Ever smoked							*p* = 0.118
Y	128	40.3	91	43.3	37	34.3	
N	190	59.7	119	56.7	71	65.7	
Ever smoked and:	*N* = 123		*N* = 89		*N* = 34		
Stopped before pregnancy	85	69.1	59	66.3	26	76.6	*p* = 0.136
Stopped when pregnant and not since	27	22.0	21	23.6	6	17.6	
Still smoke but reduced cigarettes	10	8.1	9	10.1	1	2.9	
Still smoke the same number of cigarettes as before pregnant	1	0.8	0	0	1	2.9	
E cigarette information	*N* = 302		*N* = 198		*N* = 104		
Never heard ecigs or tried them	23	7.6	17	8.6	6	5.8	*p* = 0.383
Heard of ecigs and never tried them	250	82.8	159	80.3	91	87.5	
Tried ecigs but do not use them	27	8.9	20	10.1	7	6.7	
Tried ecigs and use them	2	0.7	2	1.0	0	0	

**Table 3:** Pregnant women and mothers’ reported exposures to secondhand smoke (SHS).

	Total		Antenatal (AN)		Postnatal (PN)		Significance (*p* < 0.05)
	Frequency	%	Frequency	%	Frequency	%	
In last week exposed to SHS	*N* = 322		*N* = 212		*N* = 110		
Y	124	38.5	88	41.5	36	32.7	*p* = 0.269
N	185	57.5	115	54.2	70	63.6	
Don’t know	13	4.0	9	4.3	4	3.6	
Live with a smoker	*N* = 319		*N* = 209		*N* = 110		
Y	54	16.9	42	20.1	12	10.9	*p* = 0.038
N	265	83.1	167	79.9	98	89.1	
Relationship to smoker in the home	*N* = 51		*N* = 41		*N* = 10		
Smoker is spouse/partner	45	88.2	37	90.2	8	80.0	*p* = 0.367
Smoker is another family member	6	11.8	4	9.8	2	20.0	
Has smoke free area in the home	*N* = 307		*N* = 199		*N* = 108		
Y	287	93.5	184	92.5	103	95.4	*p* = 0.032
N	7	2.6	15	7.5	5	4.6	
Exposure to SHS indoors	*N* = 312		*N* = 203		*N* = 109		
Daily	6	1.9	5	2.4	1	0.9	*p* = 0.456
Weekly	22	7.1	16	7.9	6	5.5	
Less frequently	284	91.0	182	89.7	102	93.6	
Exposure to SHS car	*N* = 309		*N* = 201		*N* = 108		
Daily	2	0.6	1	0.5	1	0.9	*p* = 0.692
Weekly	1	0.3	1	0.5	0	0	
Less frequently	306	99.0	199	99.0	107	99.1	

**Table 4:** Support for hospital policies and services.

	Total		Antenatal (AN)		Postnatal (PN)		Significance (*p* < 0.05)
	Frequency	%	Frequency	%	Frequency	%	
Should smoking cessation support be available in hospital	*N* = 301		*N* = 195		*N* = 106		
Y	232	77.1	146	74.9	86	81.1	*p* = 0.217
N	69	22.9	49	25.1	20	18.9	
Aware smoke-free hospital	*N* = 321		*N* = 211		*N* = 110		
Y	306	95.3	202	95.7	104	4.6	*p* = 0.808
N/Don’t know	15	4.7	9	4.3	6	5.5	
Agree with smoke-free policy	*N* = 322		*N* = 212		*N* = 110		
Y	314	97.5	205	96.7	109	99.1	*p* = 0.322
N/Don’t know	8	2.5	7	3.3	1	0.1	
Does passive smoke damage health	*N* = 322		*N* = 212		*N* = 110		
Y	308	95.7	202	95.3	106	96.4	*p* = 0.327
N/Don’t know	14	4.3	10	4.7	4	3.6	

**Table 5:** Logistic regression analyses for secondhand smoke exposure.

Outcome Variable	Univariate OR	95% CI	Adjusted OR	Adjusted 95% CI	*p* Value (0.05) (Adjusted OR)
Age	0.95	0.90–1.00	0.95	0.89–1.01	0.083
Live with smoker	0.25	0.13–0.47	3.89	1.86–8.15	0.001
Highest education *	1.11	0.56–2.21	0.58	0.26–1.31	0.189
Registration status ^	0.69	0.39–1.23	1.27	0.66–2.41	0.474

* Primary/post primary/higher education. ^ Public/private. R^2^ 9.7%, Hosmer Lemeshow Test x^2^ 8.9 (df 8) 0.3.
